# Outcome of the novel description of arterial position changes after major liver resections: retrospective study

**DOI:** 10.1093/bjsopen/zrae110

**Published:** 2024-09-24

**Authors:** Sepehr Abbasi Dezfouli, Arash Dooghaie Moghadam, Philipp Mayer, Miriam Klauss, Hans-Ulrich Kauczor, De-Hua Chang, Mohammad Golriz, Arianeb Mehrabi, Katharina Hellbach

**Affiliations:** Department of General, Visceral and Transplantation Surgery, Heidelberg University Hospital, Heidelberg, Germany; Department of General, Visceral and Transplantation Surgery, Heidelberg University Hospital, Heidelberg, Germany; Department of Diagnostic and Interventional Radiology, Heidelberg University Hospital, Heidelberg, Germany; Department of Diagnostic and Interventional Radiology, Heidelberg University Hospital, Heidelberg, Germany; Department of Diagnostic and Interventional Radiology, Heidelberg University Hospital, Heidelberg, Germany; Department of Diagnostic and Interventional Radiology, Heidelberg University Hospital, Heidelberg, Germany; Department of General, Visceral and Transplantation Surgery, Heidelberg University Hospital, Heidelberg, Germany; Department of General, Visceral and Transplantation Surgery, Heidelberg University Hospital, Heidelberg, Germany; Liver Cancer Centre Heidelberg (LCCH), Heidelberg University Hospital, Heidelberg, Germany; Department of Diagnostic and Interventional Radiology, Heidelberg University Hospital, Heidelberg, Germany

## Abstract

**Background:**

After major liver resections, anatomical shifts due to liver parenchymal hypertrophy and organ displacement can happen. The aim of this study was to evaluate the impact of these anatomical changes on the main abdominal arteries (coeliac trunk and superior mesenteric artery) and on patient outcomes.

**Methods:**

All patients who underwent major liver resections (between January 2010 and July 2021) and who underwent preoperative and postoperative arterial-phase contrast-enhanced abdominal CT imaging were studied. Observed arterial position changes were classified into three groups: no position changes; class I position changes (vessel displacement with or without kinking with a vessel angle greater than 105°); and class II position changes (kinking less than or equal to 105°). The Mann–Whitney test and the Kruskal–Wallis test were used to compare continuous variables and the chi-squared test and Fisher’s exact test were used to compare categorical variables. Univariable and multivariable logistic regression analyses were used to identify the risk factors for morbidity and mortality.

**Results:**

A total of 265 patients (149 men and median age of 59 years) were enrolled. Arterial position changes were detected in a total of 145 patients (54.7%) (99 patients (37%) with class I position changes and 46 patients (18%) with class II position changes) and were observed more often after extended resection and right-sided resection (*P* < 0.001). Major complications were seen in 94 patients (35%) and the rate of mortality was 15% (40 patients died). Post-hepatectomy liver failure (*P* = 0.030), major complications (*P* < 0.001), and mortality (*P* = 0.004) occurred more frequently in patients with class II position changes. In multivariable analysis, arterial position change was an independent risk factor for post-hepatectomy liver failure (OR 2.86 (95% c.i. 1.06 to 7.72); *P* = 0.038), major complications (OR 2.10 (95% c.i. 1.12 to 3.93); *P* = 0.020), and mortality (OR 2.39 (95% c.i. 1.03 to 5.56); *P* = 0.042).

**Conclusion:**

Arterial position changes post-hepatectomy are observed in the majority of patients and are significantly related to postoperative morbidities and mortality.

## Teaser

1. Arterial position changes were detected in 55% of patients and were more usual after extended and right-sided hepatectomy.
2. Postoperative morbidities and mortality occurred more frequently in patients with severe arterial changes.
3. Arterial position change was an independent risk factor for post-hepatectomy liver failure, major complications, and mortality.

## Introduction

Major liver resection is the therapy of choice for many benign and malignant large liver tumours^1^. The development of novel perioperative diagnostic modalities and therapeutic management strategies has increased the number of major liver resections; however, these operations can lead to high rates of postoperative complications, including post-hepatectomy liver failure (PHLF) and death^2–4^. Many attempts have been made in recent decades to reduce these problems, but the results that have been achieved are still far from satisfactory, as many underlying aetiologies are still unknown^4^.

After major liver resections, the parts of the abdominal cavity no longer occupied by the liver tissue can be filled by other abdominal organs. In particular, the small and large intestine loops or the remaining parts of the liver can shift to the right upper abdomen. This relocation of the organs may result in a relocation of the supplying vascular structures^5^. As arterial blood supply is crucial, especially in the postoperative setting, and as the blood supply of the liver originates directly from the coeliac trunk and indirectly from the superior mesenteric artery (SMA) (through the pancreas head arterial arcade), the relocation of these arteries is of particular interest^6,7^. Postoperative kinking of these vessels may lead to alterations in blood flow, resulting in an impairment of the blood supply of the remnant liver, resulting in a broad variety of ischaemic complications. These changes have not been studied yet.

The aim of this study was to evaluate the effect of postoperative organ shifts on the major abdominal arteries (coeliac trunk and SMA) and to analyse the impact of these anatomical changes on the postoperative outcomes of patients after major liver resections.

## Methods

### Study design

Electronic records of patients who underwent major liver resections between January 2010 and July 2021 in the Department of General, Visceral and Transplantation Surgery of Heidelberg University Hospital were retrospectively screened for inclusion in the study (using a database containing data that were collected prospectively). Patients aged at least 18 years who underwent major liver resections, with preoperative and postoperative contrast-enhanced abdominal CT images available, were identified. The presence of a contrast-enhanced CT-scan with an arterial phase including reconstructions in the coronal and/or sagittal plane were mandatory for study inclusion. Major hepatic resection was considered as the removal of more than three liver segments, based on the Brisbane 2000 definitions. This included hemihepatectomies and extended hepatectomies (resection of more than five liver segments)^8^. This single-centre study was conducted in accordance with the Declaration of Helsinki^9^. Institutional review board approval was obtained before the study (Ethics Commission of the Medical Faculty, Ruprecht-Karls-Universität Heidelberg, Germany; reference no. S754/2018). All patients provided informed consent before discharge from the hospital.

### Patient data

#### Preoperative data

The preoperative characteristics of patients, including sex, age, body mass index (BMI), American Society of Anesthesiologists (ASA) grade^10^, and history of neoadjuvant chemotherapy and/or radiotherapy, were extracted from the database.

#### Intraoperative data

The type of resection and the side of resection were documented based on the Brisbane 2000 definitions^8^. If the triangular ligament was not left intact, the rest of the liver remnant was fixed with sutures to the falciform ligament. Blood loss and duration of operation were also obtained from the prospective database.

#### Postoperative data

Clavien–Dindo grades were used to classify surgical complications^11^. Postoperative complications greater than Clavien–Dindo grade IIIa were classified as major. Post-hepatectomy bile leakage (PHBL), post-hepatectomy haemorrhage (PHH), and PHLF were defined based on the International Study Group of Liver Surgery (ISGLS) criteria^12–14^. Relaparotomies were recorded. The durations of ICU, intermediate care unit (IMC), and hospital stays were collected from patient charts. Furthermore, 90-day mortalities were recorded.

### Radiological evaluation

CT images were independently assessed by two attending radiologists with more than 10 years of experience in abdominal imaging. The radiologists were blinded with regard to patient outcomes. In the case of discordance concerning radiological evaluation, a consensus evaluation was performed.

**Fig. 1:**
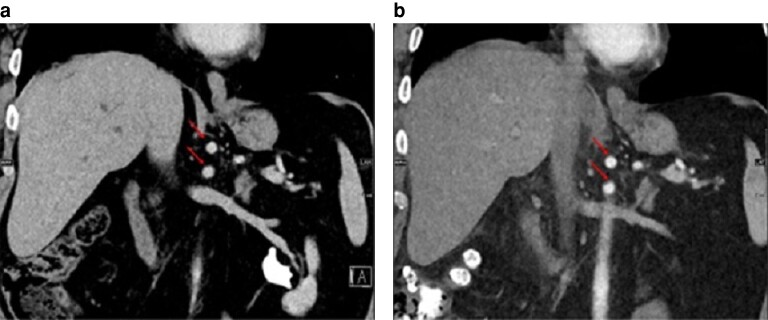
No change in position of the coeliac trunk and superior mesenteric artery (arrows) in-between the preoperative image and the postoperative image (15 days after left-sided hemihepatectomy) **a** Preoperative image. **b** Postoperative image. Coronal reconstruction.

**Fig. 2:**
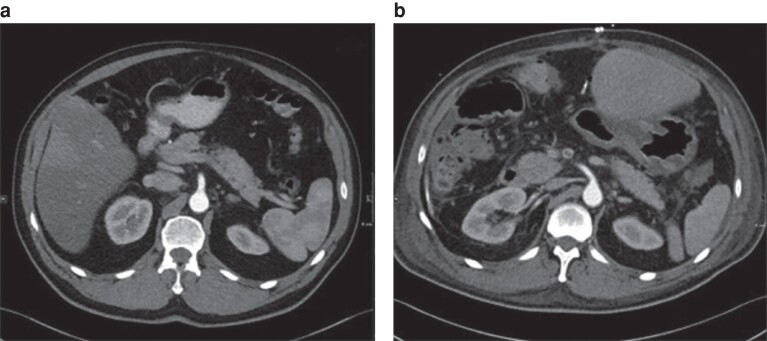
Class I position change of the superior mesenteric artery in-between the preoperative image and the postoperative scan (11 days after right-sided hemihepatectomy) **a** Preoperative image. **b** Postoperative image. Axial plane.

**Fig. 3:**
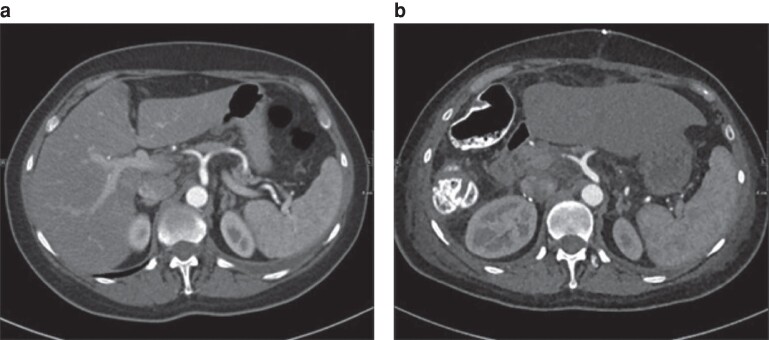
Class I position change of the coeliac trunk in-between the preoperative image and the postoperative scan (12 days after right-sided hemihepatectomy) **a** Preoperative image. **b** Postoperative image. Axial plane.

**Fig. 4:**
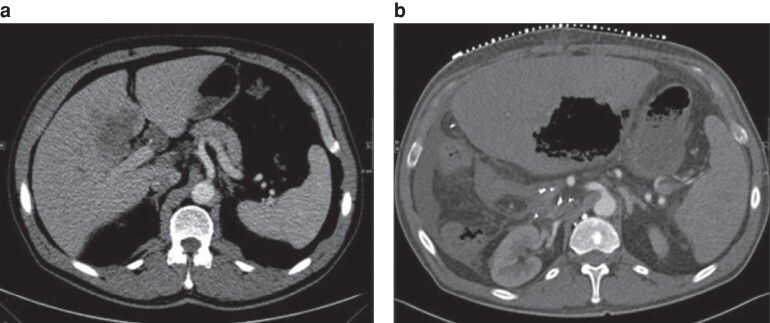
Class II position change of the coeliac trunk in-between the preoperative scan and the postoperative scan (32 days after right-sided hemihepatectomy) **a** Preoperative image. **b** Postoperative image. Axial plane. Note the gas-filled hepatic lesion in the remaining left lobe, compatible with hepatic gangrene, in the postoperative image. Postoperative imaging was performed for CT-guided drainage.

After review and comparison of preoperative and postoperative CT images from the patients’ archive, any changes in the position of the coeliac truck and SMA were assessed and divided into three groups: no position changes (*Fig. 1*); class I position changes (vessel displacement with or without kinking with a vessel angle greater than 105°; *Fig. 2* and *Fig. 3*); and class II position changes (kinking less than or equal to 105°; *Fig. 4* and *Fig. S1*). A cut-off of 105° was chosen because, starting from this angle, a stenosis was seen in the artery.

### Statistical analysis

SPSS^®^ (IBM, Armonk, NY, USA; version 25.0 for Windows) was utilized for all statistical analyses. *P* < 0.050 was considered statistically significant. Categorical variables are presented as *n* (%). Continuous variables were tested for normal distribution using the Shapiro–Wilk test. According to the non-normal distribution of the data, continuous variables are presented as median (interquartile range). The Mann–Whitney test and the Kruskal–Wallis test were used to compare continuous variables and the chi-squared test and Fisher’s exact test were used to compare categorical variables. Univariable and multivariable logistic regression analyses were used to identify independent risk factors for PHLF, major complications, and 90-day mortality. A variable with *P* < 0.200 in the univariable analysis was eligible for multivariable regression analysis. Results of regression analyses are presented as OR (95% c.i.).

## Results

### Patient characteristics

After evaluation of the preoperative and postoperative CT images, 265 patients were enrolled in this study. The baseline characteristics of the included patients are shown in *Table 1*. No patient underwent resection of the coeliac trunk and SMA, and no preoperative portal vein embolization was performed in the included patients. A total of 149 patients (56.2%) were male. Neoadjuvant therapy (radiotherapy or chemoradiotherapy or both) was given to 93 patients (35%). Hemihepatectomy was performed in 191 patients (72.1%) and the remaining patients underwent extended hepatectomy resection. Right-sided liver resection was performed in 200 patients (75.5%).

**Table 1:** Baseline characteristics and intraoperative and postoperative outcomes of patients after major liver resections

	All patients (*n* = 265)	Patients without arterial position changes (*n* = 120)	Patients with arterial position changes (*n* = 145)	P*	Patients with class I position changes (*n* = 99)	Patients with class II position changes (*n* = 46)	*P*†
**Preoperative data**							
**Sex**							
MaleFemale	149 (56.2) : 116 (43.8)	68 (57) : 52 (43)	81 (56) : 64 (44)	0.895‡	52 (53) : 47 (47)	29 (63) : 17 (37)	0.493‡
Age (years), mean(s.d.) or median (i.q.r.)	59(14)	62 (51–70)	62 (51–67)	0.722§	60 (50–67)	63 (58–67)	0.396¶
BMI (kg/m^2^), mean(s.d.) or median (i.q.r.)	25.35(4.58)	24.97 (21.83–28.04)	24.60 (22.67–27.31)	0.801§	24.9 (22.9–27.16)	24.72 (22.04–27.97)	0.934¶
**ASA grade**							
I	9 (3)	4 (3)	5 (3)	0.576#	4 (4)	1 (2)	0.500#
II	128 (48.3)	54 (45)	74 (51)		47 (48)	27 (59)	
III	94 (36)	48 (40)	46 (32)		31 (31)	15 (33)	
IV	34 (12)	14 (12)	20 (14)		17 (17)	3 (7)	
Neoadjuvant chemotherapy	91 (34)	43 (36)	48 (33)	0.641‡	34 (34)	14 (30)	0.806‡
Neoadjuvant radiotherapy	21 (8)	8 (7)	13 (9)	0.490‡	13 (13)	0 (0)	0.017‡**
Neoadjuvant therapy	93 (35)	43 (36)	50 (35)	0.819‡	36 (36)	14 (30)	0.772‡
**Intraoperative data**							
**Type of resection**							
Hemihepatectomy	191 (72.1)	94 (49)	97 (51)	0.039‡**	73 (38)	24 (13)	0.003‡**
Extended hepatectomy	74 (28)	26 (35)	48 (65)		26 (35)	22 (30)	
**Side of resection**							
Left	65 (25)	46 (71)	19 (29)	<0.001‡**	11 (17)	8 (12)	<0.001‡**
Right	200 (75.5)	74 (37)	126 (63)		88 (44)	38 (19)	
Blood loss (ml), mean(s.d.) or median (i.q.r.)	1336(1324)	950 (500–1800)	1000 (500–1600)	0.729§	1000 (500–1500)	875 (500–2000)	0.835¶
Duration of operation (min), mean(s.d.) or median (i.q.r.)	259(109)	245 (184–318)	240 (175–325)	0.955§	240 (175–322)	232 (172–349)	0.983¶
**Postoperative data**							
PHBL	86 (32)	40 (33)	46 (32)	0.781‡	26 (26)	20 (44)	0.124‡
PHH	19 (7)	9 (8)	10 (7)	0.850‡	6 (6)	4 (9)	0.817‡
PHLF	30 (11)	7 (6)	23 (16)	0.010‡**	15 (15.2)	8 (17)	0.030‡**
Relaparotomy	74 (28)	25 (21)	49 (34)	0.019‡**	26 (26.3)	23 (50)	0.001‡**
Major complications (CD grade >IIIa)††	94 (36)	32 (27)	62 (43)	0.007‡**	33 (33)	29 (63)	<0.001‡**
Duration of ICU/IMCU stay (days), median (i.q.r.)	4 (1–10)	3 (1–6)	5 (1–15)	0.001§**	4 (1–9)	9 (2–23)	0.002¶**
Duration of hospital stay (days), median (i.q.r.)	24 (14–38)	21 (13–37)	25 (15–41)	0.179§	21 (13–34)	29 (20–51)	0.012¶**
90-day mortality	40 (15)	12 (10)	28 (19)	0.035‡**	14 (14)	14 (30)	0.004‡**

Values are *n* (%) unless otherwise indicated. i.q.r., interquartile range; PHBL, post-hepatectomy bile leakage; PHH, post-hepatectomy haemorrhage; PHLF, post-hepatectomy liver failure; IMCU, intermediate care unit; CD, Clavien–Dindo. *Comparison between patients with and without arterial position changes. †Comparison between patients with different classes of arterial position changes. ‡Chi-squared test. §Mann–Whitney test. ¶Kruskal–Wallis test. #Fisher’s exact test. **Statistically significant. ††Patients may have more than one complication.

PHBL, PHH, and PHLF were observed in 86 patients (32%), 19 patients (7%), and 30 patients (11%) respectively. Major complications were seen in 94 patients (35%). In the 90-day interval after hepatectomy, 40 deaths (15%) were recorded.

The mean time interval between surgery and postoperative CT imaging was 57 (range 0–490) days. In the CT images screened, arterial position changes were detected in 145 patients (54.7%); 99 patients (37%) had class I position changes and 46 patients (17%) had class II position changes.

### Comparison of patients with and without arterial position changes

The results of the comparison of patients with and without arterial position changes are presented in *Table 1*. No significant differences were found between the two groups in terms of sex, age, BMI, ASA grade, and neoadjuvant therapy. More patients who underwent an extended hepatectomy had an arterial position change (48 *versus* 26 patients (*P* = 0.039)). Most patients who underwent a right-sided liver resection had an arterial position change (126 *versus* 74 patients (*P* < 0.001)). PHBL and PHH were found to be non-significant variables in the postoperative analysis, but PHLF, relaparotomy, and major complications occurred more frequently in patients with arterial position changes (23 *versus* 7 patients (*P* = 0.010), 49 *versus* 25 patients (*P* = 0.019), and 62 *versus* 32 patients (*P* = 0.007) respectively). The median duration of ICU/IMCU stay was longer in patients with arterial position changes (5 *versus* 3 days (*P* = 0.001)). More mortalities occurred within 90 days in the group of patients with arterial position changes (28 *versus* 12 patients (*P* = 0.035)).

### Comparison of patients with different classes of arterial position changes

The results of the comparison of patients with different classes of arterial position changes are presented in *Table 1*. Comparison of the preoperative data revealed that neoadjuvant radiotherapy was the only significant difference between the studied groups (*P* = 0.017). Hemihepatectomy was performed in the majority of patients (in 94 patients without arterial position changes and in 97 patients with arterial position changes, so in 191 patients in total). More patients who underwent a right-sided liver resection had an arterial position change in comparison with patients who underwent a left-sided liver resection (126 *versus* 74 patients (*P* < 0.001)). Analysis of the postoperative data indicated no significant differences in terms of PHBL and PHH between the studied groups. The rates of PHLF, relaparotomy, and major complications were significantly higher in patients with class II position changes (17% *versus* 15% (*P* = 0.030), 50% *versus* 26% (*P* = 0.001), and 63% *versus* 33% (*P* < 0.001) respectively). Patients with class II position changes had a longer ICU/IMCU stay compared with the other two groups (median of 9 days for patients with class II position changes *versus* 4 days for patients with class I position changes *versus* 3 days for patients without arterial position changes (*P* = 0.002)). Similarly, hospital stay was longer for patients with class II position changes compared with the other two groups (median of 29 days for patients with class II position changes *versus* 21 days for patients with class I position changes *versus* 21 days for patients without arterial position changes (*P* = 0.012)). The rate of mortality was significantly higher for patients with class II position changes compared with the other two groups (30% for patients with class II position changes *versus* 14% for patients with class I position changes *versus* 10% for patients without arterial position changes (*P* = 0.004)).

### Risk factors for post-hepatectomy liver failure, major complications, and 90-day mortality

Univariable analysis revealed that extended liver resection (*P* = 0.058), blood loss (*P* = 0.005), and arterial position changes (*P* = 0.006) were associated with PHLF (*Table S1*). In multivariable analysis, only blood loss (OR 1.00 (95% c.i. 1.00 to 1.00); *P* = 0.018) and arterial position changes (OR 2.86 (95% c.i. 1.06 to 7.72); *P* = 0.038) were independent risk factors for PHLF.

Univariable analysis revealed that male sex, age, neoadjuvant chemotherapy and radiotherapy, blood loss, duration of operation, and arterial position changes were associated with major complications (*Table S1*). In multivariable analysis, only male sex (OR 2.04 (95% c.i. 1.08 to 3.84); *P* = 0.026), age (OR 1.03 (95% c.i. 1.00 to 1.05); *P* = 0.026), neoadjuvant chemotherapy (OR 0.37 (95% c.i. 0.17 to 0.77); *P* = 0.008), and arterial position changes (OR 2.10 (95% c.i. 1.12 to 3.93); *P* = 0.020) were independent risk factors for major complications.

Univariable analysis revealed that male sex (*P* = 0.011), age (*P* = 0.001), neoadjuvant chemotherapy (*P* = 0.018), blood loss (*P* = 0.009), and arterial position changes (*P* = 0.048) were associated with 90-day mortality (*Table S1*). In multivariable analysis, only age (OR 1.05 (95% c.i. 1.01 to 1.09); *P* = 0.006), blood loss (OR 1.00 (95% c.i. 1.00 to 1.00); *P* = 0.033), and arterial position changes (OR 2.39 (95% c.i. 1.03 to 5.56); *P* = 0.042) were independent risk factors for 90-day mortality.

The association between 90-day mortality and different classes of arterial position changes was also examined (*Table 2*). Univariable analysis revealed that class II position changes were associated with 90-day mortality (*P* = 0.002). Male sex (OR 2.48 (95% c.i. 1.02 to 6.05); *P* = 0.045), age (OR 1.05 (95% c.i. 1.01 to 1.09); *P* = 0.007), neoadjuvant chemotherapy (OR 0.35 (95% c.i. 0.12 to 0.99); *P* = 0.048), blood loss (OR 1.00 (95% c.i. 1.00 to 1.00); *P* = 0.042), and class II position changes (OR 3.97 (95% c.i. 1.42 to 11.05); *P* = 0.008) were the only variables that remained significant in the multivariable analysis.

**Table 2:** Univariable and multivariable analysis of risk factors associated with major complications and 90-day mortality with a focus on arterial position changes classification

Variables	Major complications				90-day mortality			
	Univariable logistic regression		Multivariable logistic regression		Univariable logistic regression		Multivariable logistic regression	
	OR (95% c.i.)	*P*	OR (95% c.i.)	*P*	OR (95% c.i.)	*P*	OR (95% c.i.)	*P*
Male sex	2.72 (1.59,4.67)	<0.001*	2.13 (1.12,4.05)	0.020*	2.67 (1.24,5.72)	0.011*	2.48 (1.02,6.05)	0.045*
Age	1.02 (1.00,1.04)	0.010*	1.03 (1.00,1.05)	0.045*	1.05 (1.02,1.09)	0.001*	1.05 (1.01,1.09)	0.007*
BMI	1.05 (0.99,1.11)	0.073	1.04 (0.97,1.11)	0.252	1.06 (0.99,1.14)	0.090	1.08 (0.99,1.18)	0.070
ASA grade III/IV	0.70 (0.42,1.17)	0.180	0.65 (0.35,1.21)	0.173	0.85 (0.43,1.68)	0.650	–	–
Neoadjuvant chemotherapy	0.31 (0.17,0.57)	0.000*	0.333 (0.15,0.70)	0.004*	0.35 (0.15,0.84)	0.018*	0.35 (0.12,0.99)	0.048*
Neoadjuvant radiotherapy	0.27 (0.08,0.97)	0.045*	0.70 (0.17,2.85)	0.623	0.26(0.03,2.01)	0.199	0.70 (0.07,6.77)	0.756
Extended liver resection	1.70 (0.98,2.95)	0.058	1.05 (0.52,2.11)	0.885	1.48 (0.72,3.02)	0.281	–	–
Right-sided liver resection	0.92 (0.51,1.65)	0.798	–	–	1.35 (0.59–3.11)	0.471	–	–
Blood loss	1.00 (1.00,1.00)	0.001*	1.00 (1.00,1.00)	0.130	1.00 (1.00,1.00)	0.009*	1.00 (1.00,1.00)	0.042*
Duration of operation	1.00 (1.00,1.00)	0.003*	1.00 (1.00,1.00)	0.332	1.00 (0.99,1.00)	0.283	–	–
Class I position changes	1.35 (0.76,2.43)	0.301	–	–	1.48 (0.65,3.37)	0.348	–	–
Class II position changes	4.63 (2.25,9.55)	<0.001*	4.95 (2.03,12.07)	<0.001*	3.93 (1.65,9.36)	0.002*	3.97 (1.42,11.05)	0.008*

*Statistically significant. OR, Odds ratio; BMI, body mass index; ASA, American Society of Anesthesiologists.

## Discussion

In this study of 265 patients, 145 patients (54.7%) had arterial position changes after major liver resections. These changes occurred significantly more often after right-sided and extended resections, were associated with significantly higher rates of PHLF and major complications, were associated with a significantly longer hospital stay, and were associated with a significantly higher rate of mortality. Age, blood loss, and arterial position changes were independent risk factors for 90-day mortality.

This study describes the shifts and kinking of the major abdominal arteries after liver resection for the first time. It was observed that the empty space of the resected liver was filled with bowels or the remnant liver and this led to a shift in the SMA or coeliac axis with a consequent stenosis. The shifting of the liver was also due to the hypertrophy of the remnant liver in many cases. The observed radiographic changes were classified into three groups depending on the severity and this grading showed a highly relevant connection to PHLF, major complications, and mortality, with patients without arterial position changes having the lowest rates and patients with class II position changes having the highest rates.

The main known risk factors for PHLF are the volume and the function of remnant liver tissue^15–17^. The remnant liver volume is dependent on the extent of the resection. In this study, PHLF occurred more often after extended resections compared with hemihepatectomies, which is in accordance with the published literature^18^. The known factors influencing remnant liver function are history of adjuvant therapy and liver cirrhosis. However, in the present study, liver cirrhosis and adjuvant therapy were not risk factors for PHLF. On the other hand, postoperative arterial position change was an independent risk factor for PHLF. The role of arterial changes in PHLF has not been studied before. The only similar studies in this field have involved extended liver resection and split liver transplantation, suggesting portal hypertension as a factor leading to PHLF. In all these studies, the changes in blood supply were not due to the kinking or stenosis of the supplying arteries, but due to a mismatch between the higher portal inflow and the small liver remnant^19–22^. The speculated theory was that this mismatch leads to tissue shear stress and sinusoidal endothelial cell injury, which in turn result in hepatocellular damage and cell death. The observed relationship between arterial position changes and increased postoperative complications in the present study could be explained by disturbance of the blood supply, impairing liver growth and leading to PHLF. PHLF in turns leads to further complications, such as sepsis, impaired wound healing, etc.^2,23^. The findings of this study open a completely new aspect in understanding the causality of PHLF.

Based on these findings, a high-risk group of patients can be identified, including patients with one or more risk factors for complications (male sex, older age, and history of neoadjuvant chemotherapy), who undergo right-sided major or extended liver resections. In these patients, early postoperative imaging could be performed to detect the postoperative arterial changes. As a prophylactic or therapeutic strategy, arterial stent insertion could be performed to reverse the stenosis and avoid further complications. These strategies require prospective studies, which could have major impacts on avoiding and managing postoperative complications and PHLF.

There were some limitations to this study. The radiographic comparison was only possible for patients with both preoperative and postoperative images available with arterial-phase reconstructions. Including postoperative oncological follow-up imaging explains the wide range of the time of postoperative imaging. Many patients from both groups (with and without complications) were excluded due to lack of adequate preoperative or postoperative images. Therefore, selection bias is likely, but can be accepted due to the retrospective nature of the study. In some of the patients, it was not possible to differentiate between arterial kinking due to liver shift or liver hypertrophy (*Fig. S2*). Hence, it is not possible to discuss the benefit of liver fixation at the end of the operation. It was not possible to collect information regarding catecholamine therapies or other possible reasons for vasoconstrictions as a concomitant factor for stenosis.

In conclusion, changes in the coeliac trunk and SMA are seen in the majority of patients undergoing major liver resections and are associated with higher morbidity and mortality rates. Classification of these changes into three groups was suggested and was shown to have a close relationship with the postoperative results. In high-risk patients based on the performed risk analysis for complications and mortality (male sex and/or older age and/or history of neoadjuvant chemotherapy), who undergo right-sided major liver resections or extended liver resections, interventional strategies such as stent insertion could be discussed. Prospective studies are needed to confirm these data.

## Supplementary Material

zrae110_Supplementary_Data

## Data Availability

Data generated or analysed during this study are available from the corresponding author on request.
