# Supplementary material for: Outcome of the novel description of arterial position changes after major liver resections: retrospective study
Source: BJS Open. 2024 Sep 24;8(5):zrae110. doi: 10.1093/bjsopen/zrae110 (PMC11421472; doi:10.1093/bjsopen/zrae110)
Supplement: zrae110_Supplementary_Data [file zrae110_supplementary_data.docx]

**Title**

**Outcome of the novel description of Arterial position Changes after Major Liver Resections: retrospective study**

Authors

Sepehr Abbasi Dezfouli^1^, MD; Arash Dooghaie Moghadam^1^, MD; Philipp Mayer^2^, MD; Miriam Klauss^2^, MD; Hans-Ulrich Kauczor^2^, MD; De-Hua Chang^2^, MD; Mohammad Golriz^1^, MD; Arianeb Mehrabi^1,3^, MD; Katharina Hellbach^2^, MD

^1Department of General, Visceral and Transplantation Surgery, Heidelberg University Hospital, Heidelberg, Germany^

^2Department of Diagnostic and Interventional Radiology, Heidelberg University Hospital, Heidelberg, Germany^

^3 Liver Cancer Centre Heidelberg (LCCH), Heidelberg University Hospital, Heidelberg, Germany^

**Corresponding author.** Katharina Hellbach, MD, Department of Diagnostic and Interventional Radiology, Heidelberg University Hospital, Im Neuenheimer Feld 420, 69120 Heidelberg, Germany. Tel: +4962215638741

E-Mail: [Katharina.AbbasiDezfouli@med.uni-heidelberg.de](mailto:Katharina.AbbasiDezfouli@med.uni-heidelberg.de) **0009-0003-8731-2364**

**Supplementary Materials - Index**

| **Supplementary Methods** |  |
| --- | --- |
| None |  |
| **Supplementary Results** |  |
| None |  |
| **Supplementary Appendixes** |  |
| None |  |
| **Supplementary Figures and Tables** |  |
| Table S1 | *pag. 3* |
| Figure S1  Figure S2 | *pag. 5*  *pag. 6* |
| **References** | *pag. 10* |
| None |  |

**Supplementary Figures and Tables**

**Table S1. Univariable and multivariable analysis of risk factors associated with post-hepatectomy liver failure, major complications and 90-day mortality with a focus on arterial position changes.**

|  | **Post-hepatectomy liver failure** | | | | **Major complications** | | | | **90-day mortality** | | | |
| --- | --- | --- | --- | --- | --- | --- | --- | --- | --- | --- | --- | --- |
| **Variables** | **Univariable logistic**  **regression** | | **Multivariable logistic**  **regression** | | **Univariable logistic**  **regression** | | **Multivariable logistic**  **regression** | | **Univariable logistic**  **regression** | | **Multivariable logistic**  **regression** | |
|  | **OR**  **(95% CI)** | ***P*-value** | **OR**  **(95% CI)** | ***P*-value** | **OR**  **(95% CI)** | ***P*-value** | **OR**  **(95% CI)** | ***P*-value** | **OR**  **(95% CI)** | ***P*-value** | **OR**  **(95% CI)** | ***P*-value** |
| **Male gender** | **1.39 (0.63-3.06)** | **0.406** |  |  | **2.72 (1.59-4.67)** | **<0.001** | **2.04 (1.08-3.84)** | **0.026** | **2.67 (1.24-5.72)** | **0.011** | **2.38 (0.99-5.70)** | **0.052** |
| **Age** | **1.03 (0.99-1.06)** | **0.069** | **1.026 (0.99-1.06)** | **0.154** | **1.02 (1.00-1.04)** | **0.010** | **1.03 (1.00-1.05)** | **0.026** | **1.05 (1.02-1.09)** | **0.001** | **1.05 (1.01-1.09)** | **0.006** |
| **BMI** | **0.99 (0.90-1.08)** | **0.857** |  |  | **1.05 (0.99-1.11)** | **0.073** | **1.04 (0.97-1.11)** | **0.246** | **1.06 (0.99-1.14)** | **0.090** | **1.08 (0.99-1.17)** | **0.082** |
| **ASA classification III/IV** | **0.79 (0.37-1.71)** | **0.564** |  |  | **0.70 (0.42-1.17)** | **0.180** | **0.63 (0.32-1.24)** | **0.182** | **0.85 (0.43-1.68)** | **0.650** |  |  |
| **Neoadjuvant chemotherapy** | **0.80 (0.35-1.82)** | **0.596** |  |  | **0.31 (0.17-0.57)** | **<0.001** | **0.37 (0.17-0.77)** | **0.008** | **0.35 (0.15-0.84)** | **0.018** | **0.36 (0.13-1.02)** | **0.055** |
| **Neoadjuvant radiotherapy** | **0.81 (0.18-3.67)** | **0.787** |  |  | **0.27 (0.08-0.97)** | **0.045** | **0.60 (0.15-2.41)** | **0.473** | **0.26 (0.03-2.01)** | **0.199** | **0.56 (0.57-5.49)** | **0.618** |
| **Extended liver resection** | **2.18 (1.00-4.75)** | **0.050** |  |  | **1.70 (0.98-2.95)** | **0.058** | **1.33 (0.66-2.64)** | **0.423** | **1.48 (0.72-3.02)** | **0.281** |  |  |
| **Right liver resection** | **2.27 (0.76-6.79)** | **0.139** | **2.019 (0.55-7.33)** | **0.286** | **0.92 (0.51-1.65)** | **0.798** |  |  | **1.35 (0.59-3.11)** | **0.471** |  |  |
| **Blood loss** | **1.00 (1.00-1.00)** | **0.005** | **1.00 (1.00-1.00)** | **0.018** | **1.00 (1.00-1.00)** | **0.001** | **1.00 (1.00-1.00)** | **0.121** | **1.00 (1.00-1.00)** | **0.009** | **1.00 (1.00-1.00)** | **0.033** |
| **Operation time** | **1.00 (0.9-1.00)** | **0.272** |  |  | **1.00 (1.00-1.00)** | **0.003** | **1.00 (1.00-1.00)** | **0.352** | **1.00 (0.99-1.00)** | **0.283** |  |  |
| **Arterial position changes** | **3.64 (1.43-9.23)** | **0.006** | **2.86 (1.06-7.72)** | **0.038** | **1.93 (1.15-3.26)** | **0.013** | **2.10 (1.12-3.93)** | **0.020** | **2.07 (1.00-4.29)** | **0.048** | **2.39 (1.03-5.56)** | **0.042** |

**BMI: Body mass index, ASA: American Society of Anaesthesiologists.**

**
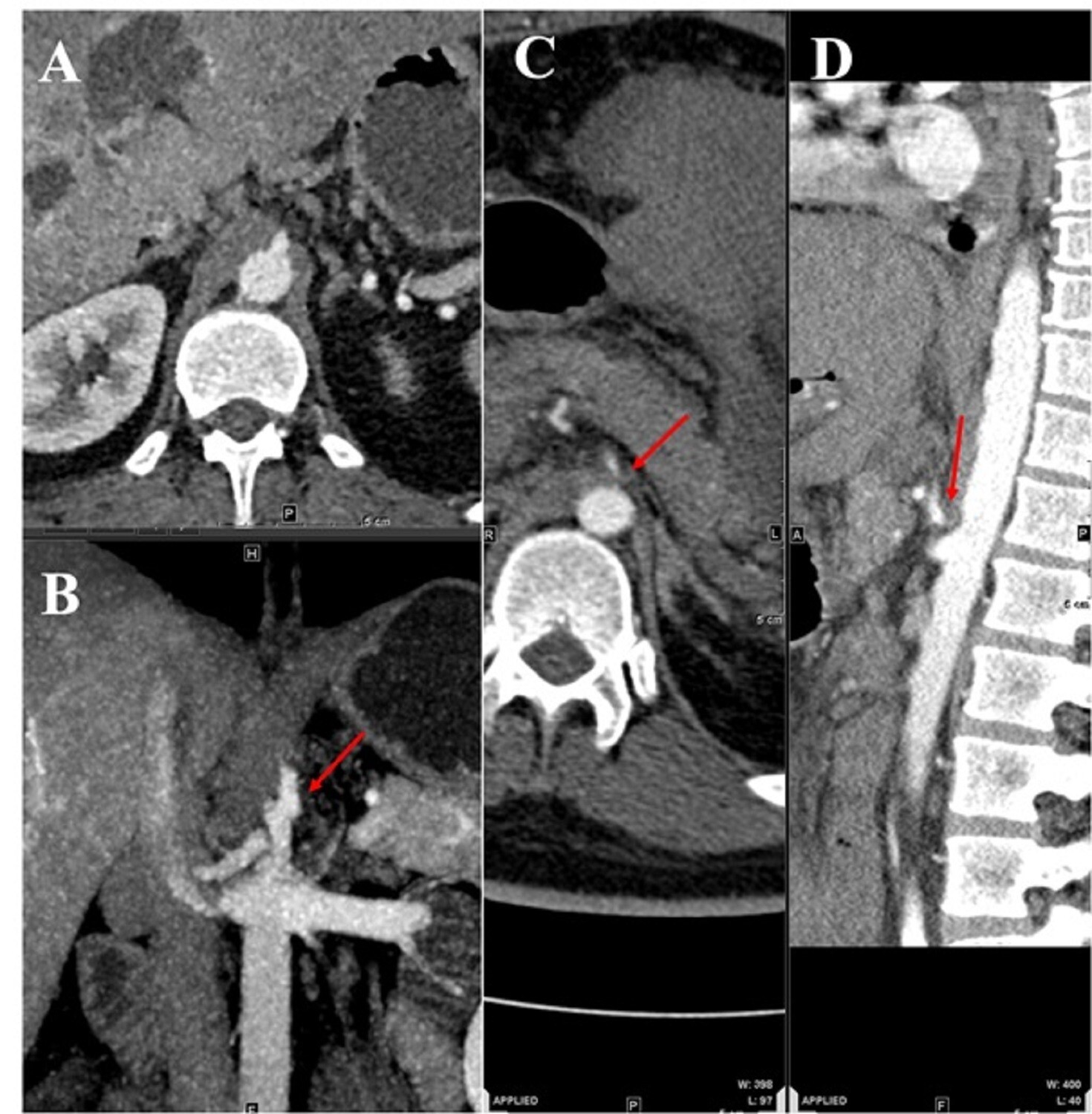
Figure S1. Position change class II. Before right-sided hemi hepatectomy (A, B) the coeliac trunk (red arrow) is rather thick and shows a caudal course (B, coronal maximal intensity projection). Four days after right sided hemi hepatectomy (C, D) there is a proximal coeliac trunk stenosis of more than 90% due to severe cranial shift of the vessel (angle 82°, D).**

**
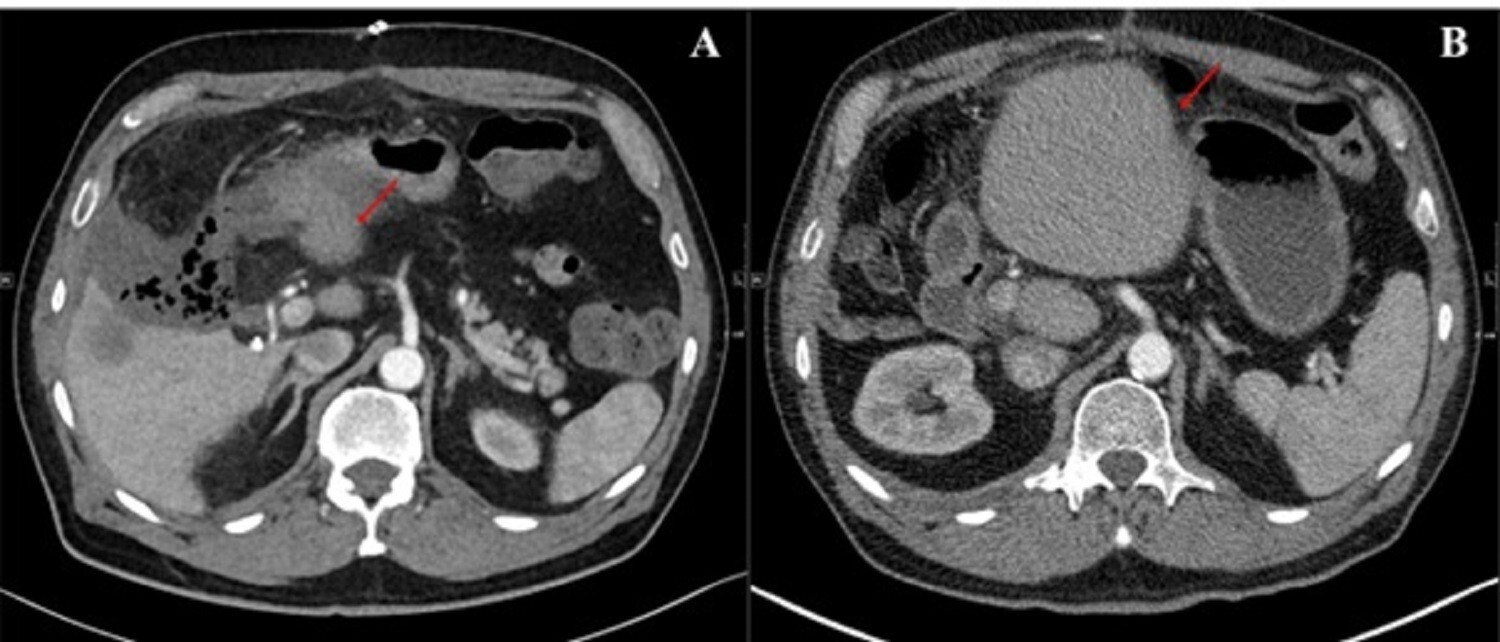
**

**Figure S2. Hypertrophy of the remaining left lobe (arrow) 72 days after right sided hemi hepatectomy (B). Position change grade I of the coeliac trunk.**
